# Meta-model of Human Recognition-behavioral Adaptation System

**DOI:** 10.1007/s12124-023-09781-0

**Published:** 2023-05-31

**Authors:** Yutaka Masuda

**Affiliations:** Psychosomatic Division, Luna Mental Clinic of Jinseikai Medical Corporation, Tsuchizaki-minato chu-ou 1-21-36, Akita, 011-0946 Japan

**Keywords:** Complex-network, Functional module, Human recognition-behavioral adaptation-system, Meta-model, Scientific modeling

## Abstract

Scientific modeling is a syllogistic system of definitive premise, sound inference and consistent explanation to understand, define, quantify, visualize or simulate feature of the target. Single-model is defined to an informative representation for identifying a property of a target object/phenomenon, and meta-model integrates the relevant single-models to explain phenomenological realities. Human recognition-behavioral adaptation is an information-metabolism system to maintain homeostasis of human-self, and that has been investigated in neurological, psychiatric and psychological aspects. I analyzed human recognition-behavioral adaptation-system via scientific modeling. Neurological meta-model of human recognition-behavioral adaptation system was synthesized as complex-network of the functional neuronal modules, and the meta-model was integrated to Mentality-model in the psychiatric aspect, and to Personality-model in the psychological aspect. The integrated meta-models successfully explained phenomenological realities in the aspects. From the above, I comprehended that the meta-model of human recognition-behavioral adaptation-system has been developed to Biopsychosocial model integrating the biological, psychological and socio-environmental factors.

## Introduction

Science is a thinking-procedure to organize the target in the form of testable explanations via logical reasoning, and scientific modeling is performed to understand, define, quantify, visualize or simulate feature of the target by referencing existing and commonly accepted knowledge. Single-model is defined to an informative representation for identifying single-property of target object/phenomenon, and meta-model integrates the relevant single-models to explain phenomenological realities. *Human recognition-behavioral adaptation-system* consists of the multiple properties, and the system has been investigated in Neurology, Psychiatry and Psychology. In this paper, I integrate the meta-model of *human recognition-behavioral adaptation-system* to explain the neurological, psychiatric and psychological realities.

## Scientific Modeling

### Structure of Scientific Modeling System

Scientific procedure is performed via syllogistic hierarchy of definitive premise, sound inference and consistent explanation. Inference consists of qualitative-reasoning induction and quantitative-reasoning deduction. Analogy is induction of homology and simplification, Pattern-recognition is induction/deduction of clustering, and Algorism is deduction of calculation. *Scientific modeling* is performed under premising via analogy, constructing model via pattern-recognition and explaining phenomenological realities via algorism. Abduction gives valid explanations to phenomenological realities in the different aspects via the model-integration.

### Single-model and Meta-model

Single property of a target object/phenomenon is resulted in a single-model of proposition and/or formula via *scientific modeling*. Multiple properties of the target are organized to a *meta-model* represented as *complex-network* of the relevant single-models. Validity of the individual single-model is assessed by Correlation-analysis. Validity of the organized meta-model that could be assessed by Multiple-regression-analysis, is usually assessed by actually-explaining phenomenological realities.

## The Neurological Meta-model

### The Model-organization

**Fig. 1:**
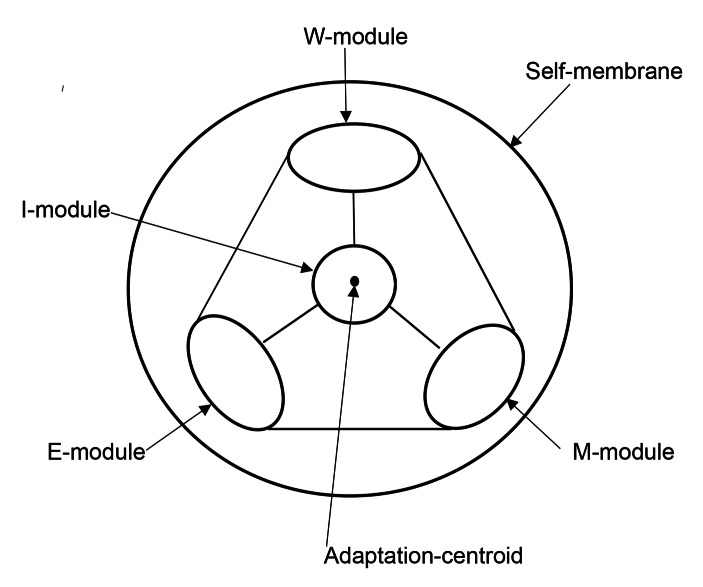
Neurological Meta-model The meta-model is constructed as complex-network of cholinergic module, adrenergic module, serotonergic module and dopaminergic module. The cholinergic module is defined to M (Memory)-module preserving stress-coping memories, the adrenergic module is defined to W (Will)-module inducing stress-coping behaviors, the serotonergic module is defined to E (Emotion)-module keeping physical strength, and the dopaminergic module is defined to I (Integration)-module integrating these module-functions. I-module places at Adaptation-centroid of MEW-tringle because of the role maintaining adaptation-integrity. The module-size expresses the activity. The meta-model is covered by Self-membrane

Human recognition-behavioral adaptation is defined to information-processing system to maintain homeostasis of human-self, and premise of the meta-model is created with knowledge of Neuroscience. Neuroscientific studies reported that mammalian brain mainly works via *complex-network* of the *adrenergic, serotonergic, cholinergic* and *dopaminergic modules* (Bullmore & Sporn, 2012; Perona et al., 2008; Sarnyai and Kovacs 1994). Complex-network of the neuronal modules is considered to the neurological meta-model of *human recognition-behavioral adaptation-system*. The serotonergic module is defined to E (Emotion)-module, the adrenergic module is to W (Will)-module, the cholinergic module is to M (Memory)-module, and the dopaminergic module is to I (Integration)-module. I-module places in centroid of MEW-triangle because of the integration-role, and the centroid is distinguished to Adaptation-centroid. Lately, human humoral stress-coping glycolipids was reported to follow the module-activities (Masuda, 2020). Namely, function of the module is epigenetically determined, and the activity is represented as the module-size corresponding to the glycolipid-production. Neurological meta-model is symbolically schemed in (Fig. 1).

### The Phenomenological Explanation

W-M-I circuit induces conditionings, M-E-I circuit induces emotional behaviors, and E-W-I circuit induces instinctive behaviors. I-(M-E-W) circuit induces voluntary learning to gain successful stress-coping. I-module recognizes success of the stress-coping as increase of the pleasure and/or decrease of the discomfort. Depressive-state is related with activities of E-module, Manic-state is related with W-module activity, and Hallucination and Delusion are related with I-module activity.

## Mentality-model

### The Model-organization

**Fig. 2:**
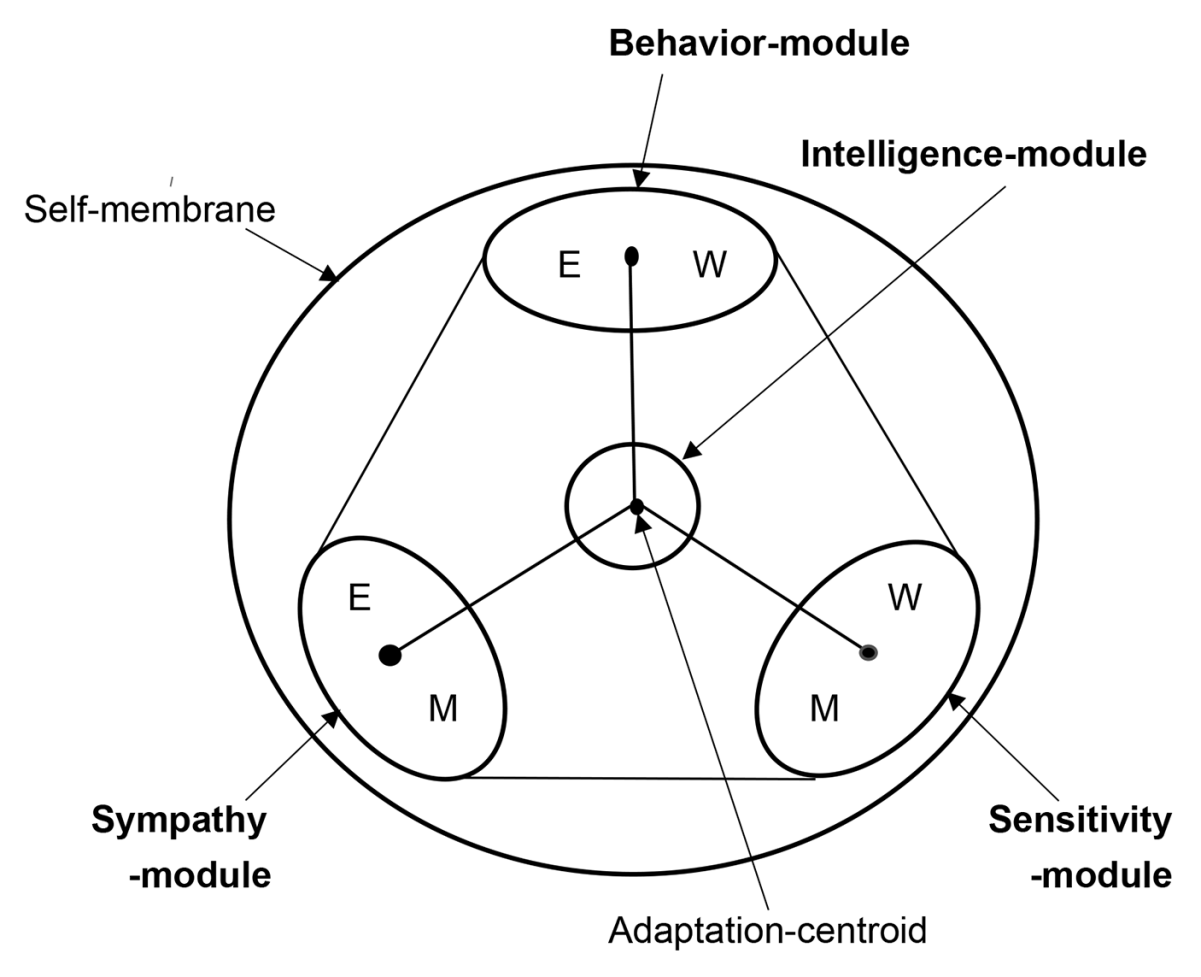
Mentality-model M-module and W-module of the neurological meta-model are integrated to Sensitivity-module. Sensitivity-module is responsible for the stress-sensitivity inducing emotional behavior. W-module and E-module of the neurological meta-model are integrated to Behavior-module. Behavior-module is responsible for the social behavior induced by conditioning. E-module and M-module of the neurological meta-model are united to Sympathy-module. Sympathy-module is responsible for the social allies-recognition relating with instinct behavior. I-module of the meta-neurological model has evolved to Intelligence-module corresponding to acquirement of the language-operation. Intelligence-module integrates information-input from the other modules

Mentality is considered to the complex of sensitivity, behavior, emotion and intelligence. M-module and W-module are integrated to Sensitivity-module which is responsible for the stress-sensitivity inducing instinct behavior. Sensitivity-module involves exceed stimulation-amplify of Autism-Spectrum Disorder (ASD) patients (Tarver et al., 2020). W-module and E-module are integrated to Behavior-module which is responsible for the social behavior induced by conditionings. Behavior-module involves hyperactivity and inattention of Attention-Deficit Hyper-Activity Disorder (ADHD) patients (Keulers & Hurks, 2021). E-module and M-module are integrated to Sympathy-module which is responsible for the social allies-recognition inducing emotional behavior. Sympathy-module involves communication-disorder of ASD (Tarver et al., 2020). Intelligence-module has evolved from I-module with improvement of the language-operation. Intelligence-module integrates information-input from Sensitivity-module, Behavior-module and Sympathy-module. Intelligence-module involves Intellectual Disabilities and Specific Learning Disorders of Dyslexia, Dysgraphia and Dyscalculia (Fletcher et al., 2005). This is Mentality-model. Mentality-model is symbolically schemed in (Fig. 2).

### The Phenomenological Explanation

Sensitivity-module that is mainly regulated with cholinergic neuron and adrenergic neuron, works to control circumstance-information input. However, the stimulation-amplify is mediated by mood stabilizers of lithium and anti-epileptics which block Na-channels of neuron-membrane. Behavior-module that is mainly regulated with adrenergic neuron and serotonergic neuron, works to condition fight-behavior and flight-behavior. In fact, hyperactivity and inattention of ADHD is treated with agents affecting alpha2A-aderenoceptor and 5HT receptor (Arnsten, 2006; Rastmanesh, 2010). Sympathy-module that is mainly regulated with serotonergic neuron and cholinergic neuron, works to communicate the sympathy-allies. Nevertheless, drags treating the communication-disorder have not been found. Intelligence-module that is mainly regulated with dopaminergic neuron, works to maintain consistency of the integration via language-usage. Nevertheless, drags treating Intellectual Disabilities and Specific Learning Disorders have not also been found. Now, amplified circumstance-information input induces discomfort. When Intelligence-module has identified origin of the discomfort, Behavior-module expresses fight behavior and/or flight behavior to deal with the origin. Failure of the fight results in Depression, and failure of the flight results in Substance-abuse, Love affair exceeding, Over-eating and/or Self-injury, which induce quick and temporary pleasure via dopaminergic neuronal stimulation. After exposed serious discomfort for a long-time, human might try to commit Suicide to eliminate the discomfort. When Intelligence-module has not identified origin of the discomfort, the mentality-system recognizes Anxiety. Behavior-module induces Stereotyped-behavior to decrease Anxiety, and Intelligence-module induces Hallucination and/or Delusion to explain origin of Anxiety. Medication for Hallucination and/or Delusion has been performed with anti-dopaminergic agents, however, medication for Stereotyped-behavior is very difficult. On the other hand, Somatization and Pain that are induced by a psychological problem could be improved by several atypical antipsychotics and/or an anti-epileptic agent carbamazepine.

## Personality-model

### Structure of The Module

**Fig. 3:**
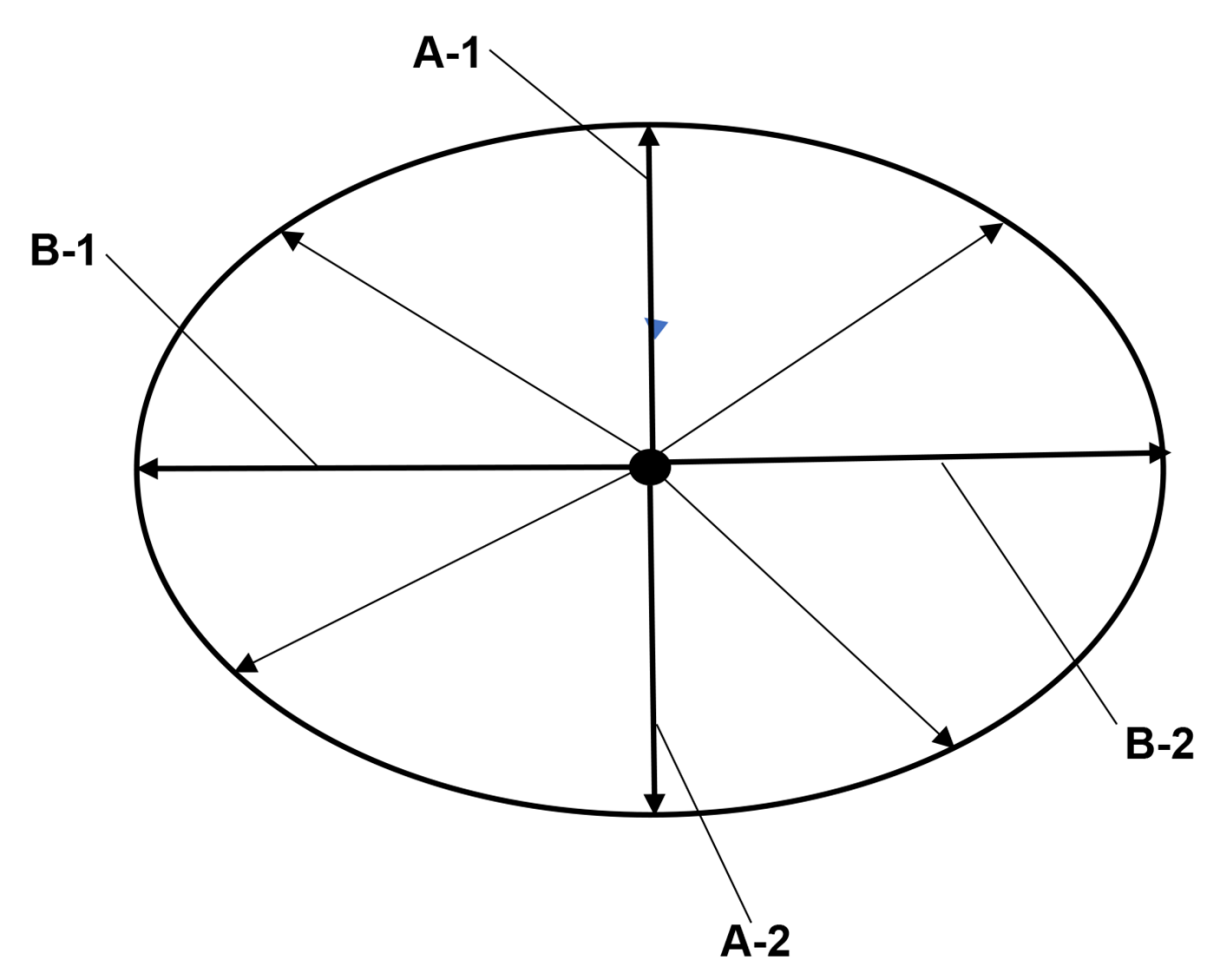
Module-structure of Personality-model Sensitivity-module is integrated to two-dimensional vector-spectrum space of (A1-A2) x (B1: M-module-function - B2: W-module-function). Behavior-module is integrated to two-dimensional vector-spectrum space of (A1: direction-for-others - A2: direction-for-self) x (B1: W-module-function - B2: E-module-function). Sympathy-module is integrated to two-dimensional vector-spectrum space of (A1-A2) x (B1: E-module-function - B2: M-module-function)

Personality is considered to psychological aspect of Mentality. Researchers have investigated psychometrical provisions via questionnaires of Big Five personality traits. The traits are distinguished as Extroversion, Openness, Conscientiousness, Agreeableness, and Neuroticism (Rothmann & Coetzer, 2003). These personality-traits were further analyzed with the Structure of Temperament Questionnaire via Lexical hypothesis. The hypothesis comes from the idea that an individual personal feature is expressed as a single word (Trofimova, 2014). The questionnaire was simply arranged to Ten-Item Personality Inventory (TIPI) (Nues et al. 2018), however, TIPI represented many sub-types even via Machine Learning (Soutter et al., 2020). Nevertheless, results of TIPI would be exchanged to a two-dimensional vector-spectrum (Neuman & Cohen, 2014). Namely, Sensitivity-module is integrated to two-dimensional vector-spectrum space of (A1: direction-for-others - A2: direction-for-self) x (B1: M-module-function - B2: W-module-function). The A1 directs to the positive approval/estimation of the belonging society, and the A2 directs to the positive approval/estimation of the self. Neuroticism is involved in Sensitivity-module. Behavior-module is integrated to two-dimensional vector-spectrum space of (A1-A2) x (B1: W-module-function - B2: E-module-function). The A1 directs to fight-attitude forward the society, and the A2 directs to flight-attitude from the society. Openness and Extroversion are involved in Behavior-module. Sympathy-module is integrated to two-dimensional vector-spectrum space of (A1-A2) x (B1: E-module-function - B2: M-module-function). The A1 directs to rules of the belonging society, and the A2 directs to rules of the individual relationships. Conscientiousness and Agreeableness are involved in Sympathy-module. Function-quantity of the module is represented as the module-size, and the function-quality is represented as the module-shape. Structure of the module is schemed in (Fig. 3).

### Standard-shape of Personality

**Fig. 4:**
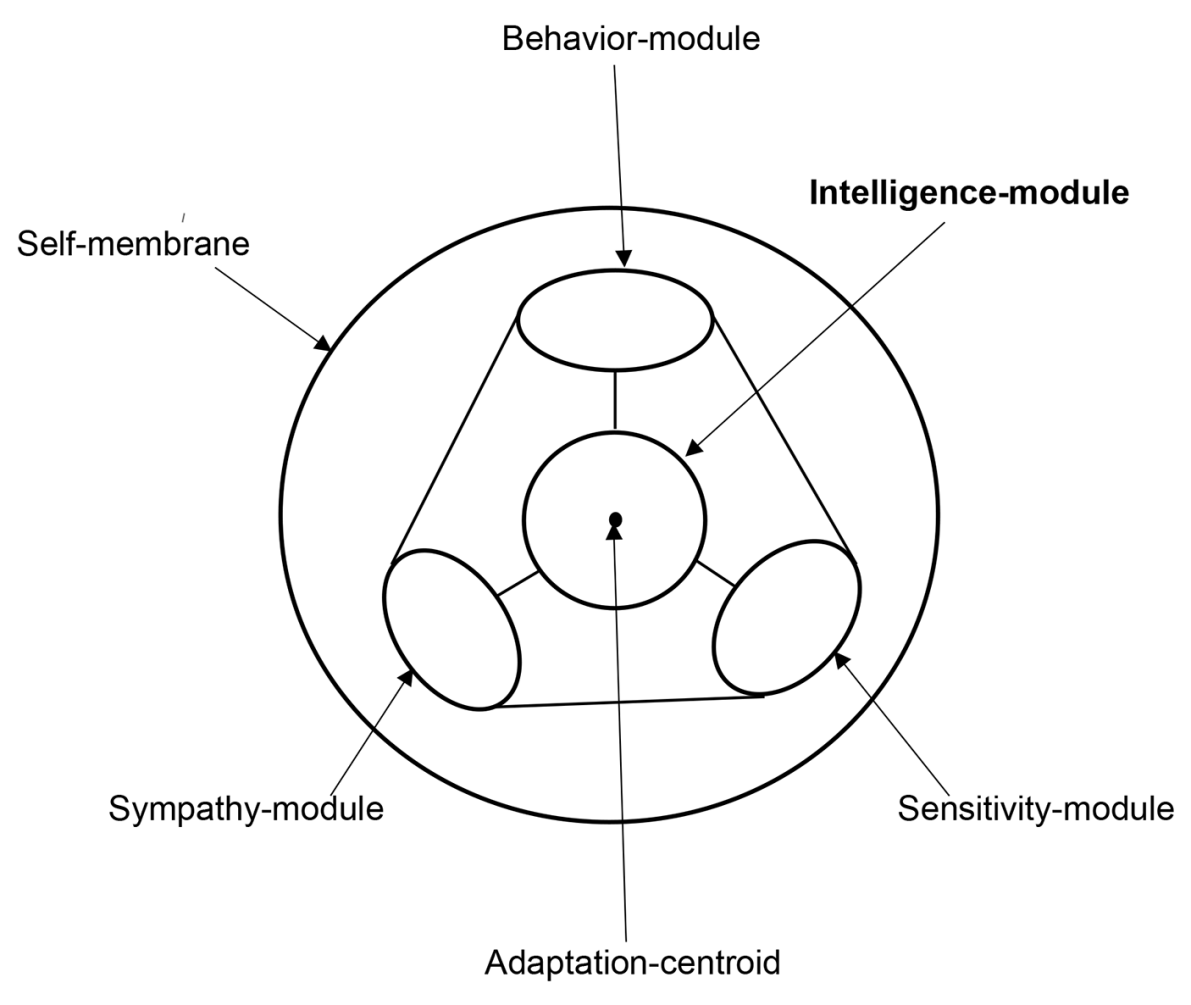
Standard-shape of Personality Sensitivity-module involves Neuroticism. Behavior-module involves Openness and Extroversion. Sympathy-module involves Conscientiousness and Agreeableness. These modules are not small nor deformed

Human maintains the social adaptation by getting positive approval/estimation of sexual charm, finance and hierarchy. The positive evaluation induces pleasure, and the negative evaluation induces discomfort. Sensitivity-module works to amplify the evaluation-input. Human maintains the social adaptation with social behaviors coming from fight and flight. Exceeding fighting results in Anti-social behavior, and exceeding flight results in Social withdraw. Behavior-module works to adequately-condition the social behaviors. Human maintains the social adaptation by imitating social adaptation-model. Sympathy-module works to communicate the model with sympathy-sense. Society has a personality-standard corresponding to the majority. Standard-shape of personality is symbolically schemed in (Fig. 4). Social stress of the individual is less in the group whose personality-shape is homologous.

### Deformed Personality-shape

**Fig. 5:**
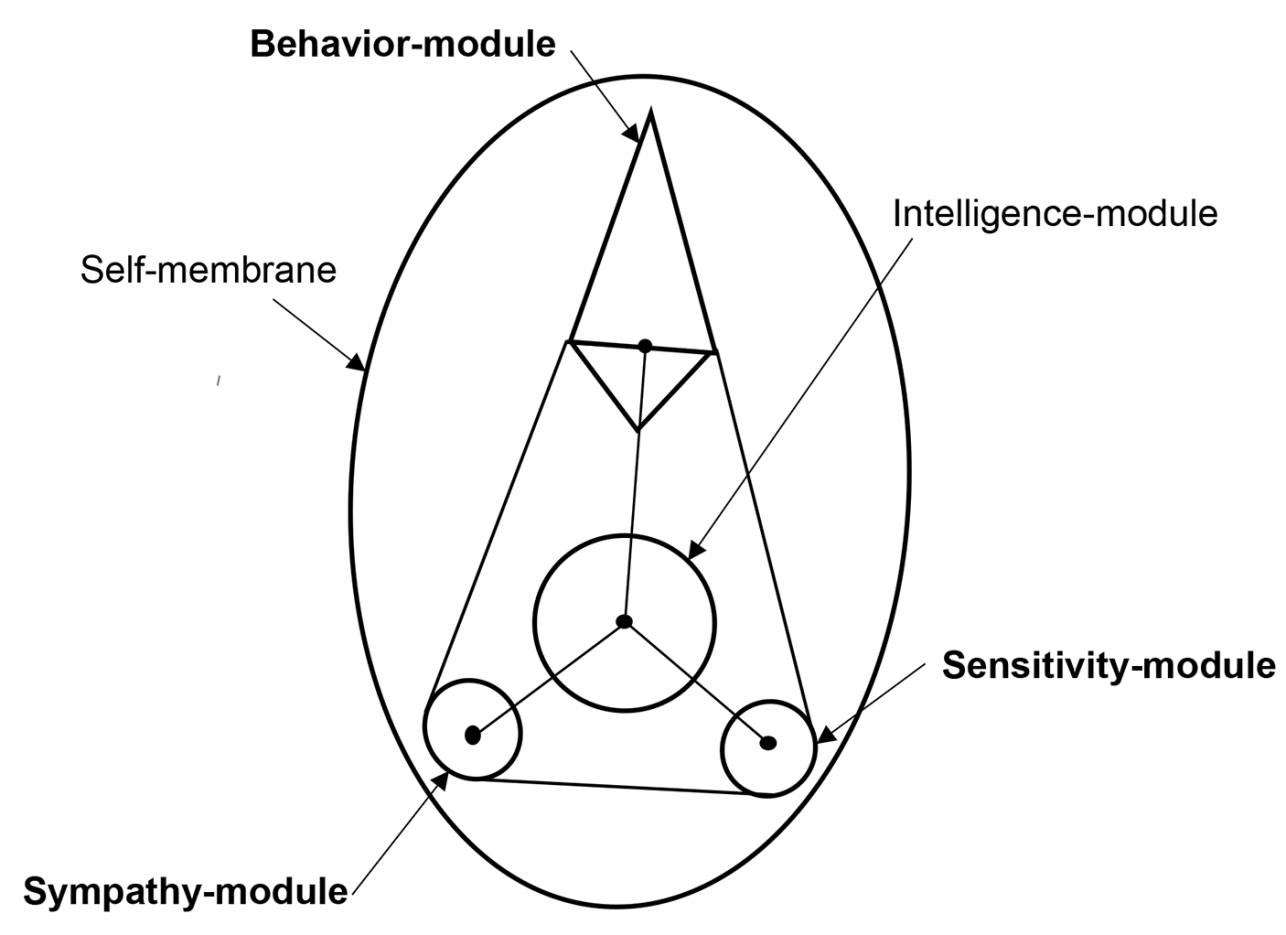
Shape of Evil Personality Evil Personality is characterized with Dark Triads: Narcissism; Machiavellianism; Psychopath. Common attitudes of these characteristics are decrease of Conscientiousness and Agreeableness, and increase of Extroversion. So, Evil Personality is represented as the small-sized Sympathy-module, the small-sized Sensitivity-module and A1-direction-elongation of the distorted large-sized Behavior-module. The shape is very different from shape of standard personality, and that is compatible to shape of Highly Sensitive Person

When the standard of personality-configuration is recognized, some persons having the special personality-configuration are distinguished. They have superior-functional modules or inferior-functional modules. Person having a superior-functional module would be considered as a fascinating heretic. Person of superior Sensitivity-module is sensitive to the social standard of approval/estimation. Person of superior Behavior-module is erotic, and has brutal vitality called Libido. Person of superior Sympathy-module behaves with a philanthropic manner. Person of superior Intelligence-module would produce original inventions. Person who has superior Intelligence-module and superior Sensitivity-module, is known as Intellectual Gifted (Mendaglio, 2010). Person who has superior Sympathy-module and superior Sensitivity-module, is called to worrying Highly Sensitive Person (Montoya-Pérez et al., 2019). Person who has superior Sensitivity-module and superior Behavior-module, might be diagnosed to Borderline Personality-disorder. They are annoyed with the amplified information-input, and often control the interested persons with anti-social behavioral manner (Bozzatello et al., 2021). Person having an inferior-functional module is considered as Personality-disorder. The person has an individual recognition-behavioral adaptation-strategy to compensate for the module-inferiority. Deviation of Intelligence-module is related with Paranoid, Schizoid or Schizotypal Personality-disorder, and the strategy is delusion-forming to keep the thinking-consistency. Deviation of Sensitivity-module is related with Histrionic Personality-disorder, and the strategy is behavioral decoration to get the social positive approval/estimation. The deviation is also related with Narcissistic Personality-disorder, and the strategy is self-satisfaction to keep the absolute self-estimation. Deviation of Behavior-module is related with Avoidant Personality-disorder, and the strategy is social flight to avoid struggle in the belonging society. This deviation is also related with Obsessive-Compulsive Personality-disorder, and the strategy is stereotypic behaviors to decease the uneasiness. Deviation of Sympathy-module is related with Dissociative Personality-disorder, and the strategy is dissociation/conversion to psychologically-protect the self. Evil Personality has been recognized as personality of Dark Triads (Furnham et al., 2013). Dark Triads is Narcissism, Machiavellianism and Psychopathy. These characteristics were also investigated by using TIPI (Paulhus & Williams, 2002). Main attitude of Narcissism is increase of Extroversion and Openness, that of Machiavellianism is decrease of Conscientiousness and Agreeableness, and that of Psychopathy is decrease of Conscientiousness and Agreeableness and increase of Extroversion (Vernon et al., 2008). Namely, Evil Personality is represented as the inferior Sympathy-module, the inferior Sensitivity-module and A1-elongation of the deformed superior Behavior-module (Fig. 5).

## Discussion

Researchers have tried modeling Mentality. The great founder of Psychoanalysis Sigmund Freud represented the famous graphical mentality-model of Ido, Ego and Super-Ego which comes from floating-iceberg metaphor. “Maslow’s hierarchy of needs” was represented as a graphical mentality-model of Humanistic psychology. Behaviorism that has developed by investigating animal behaviors, pointed-out a significant property conditioning/learning in the mentality-model. Nevertheless, these models treat single-property of Mentality. On the other hand, Biopsychosocial model is a trans-disciplinary meta-model of biology, psychology and socio-environmental factors, and the constructing policy is total analysis of human recognition-behavioral adaptation. From the above, I comprehended that the researchers have phenomenologically developed my idea of human recognition-behavioral adaptation-system.

## Data Availability

Not applicable.
